# Lipidomic Profiling of Dechorionated Fertilized Eggs and Egg Chorion in Three Tropical Marine Fish Species: Insights into Reproductive Physiology and Nutrition

**DOI:** 10.3390/biology15020172

**Published:** 2026-01-17

**Authors:** Yi-Hong Liu, Hua-Yang Guo, Bao-Suo Liu, Teng-Fei Zhu, Lin Xian, Nan Zhang, Ke-Cheng Zhu, Jian-She Zhang, Dian-Chang Zhang

**Affiliations:** 1 National Engineering Research Center for Marine Aquaculture, Zhejiang Ocean University, Zhoushan 316022, China; liuyihong0223@163.com; 2 Key Laboratory of South China Sea Fishery Resources Exploitation and Utilization, Ministry of Agriculture and Rural Affairs, South China Sea Fisheries Research Institute, Chinese Academy of Fishery Sciences, Guangzhou 510300, China; guohuayang198768@163.com (H.-Y.G.); liubaosuo343@163.com (B.-S.L.); zhtfsuper@outlook.com (T.-F.Z.); xlinxlxl@163.com (L.X.); 398730316@163.com (N.Z.); zkc537@163.com (K.-C.Z.); 3 Guangdong Provincial Engineer Technology Research Center of Marine Biological Seed Industry, Guangzhou 510300, China; 4 Sanya Tropical Fisheries Research Institute, Sanya 572018, China; 5 Shenzhen Base of South China Sea Fisheries Research Institute, Chinese Academy of Fishery Sciences, Shenzhen 518121, China; 6 Guangdong Provincial Key Laboratory of Fishery Ecology and Environment, Guangzhou 510300, China; 7 Guangdong Engineering Research Center of Key Technologies and Equipment R&D on Modern Marine Ranching, Guangzhou 510300, China

**Keywords:** egg quality, maternal lipid allocation, lipid-class partitioning, glycerophospholipid metabolism, sphingolipid signaling, tropical marine aquaculture

## Abstract

Broodstock nutrition is a key determinant of reproductive output in marine fishes because lipids support yolk formation, embryonic development, and early larval viability. However, the allocation of lipid classes between fertilized eggs and the egg envelope (chorion) remains poorly characterized for many tropical species. In this study, we performed a comparative lipidomic analysis of dechorionated fertilized egg contents and isolated chorion from three tropical marine fishes (*Trachinotus ovatus*, *Platax teira*, and *Plectropomus leopardus*) using UHPLC–Q Exactive Orbitrap MS/MS. Multivariate analyses revealed clear tissue- and species-specific lipid patterns. Dechorionated eggs were enriched in energy-storage lipids, dominated by triacylglycerols and essential polyunsaturated fatty acids, whereas chorion tissues contained higher levels of structural and signaling lipids, including phosphatidylinositols and sphingolipids. Each species exhibited a distinct lipid signature, with *T. ovatus* characterized by higher secosteroids and free fatty acids, *P. teira* by glycerophosphoethanolamines and phosphoinositols, and *P. leopardus* by abundant triradylglycerols. Pathway enrichment highlighted glycerophospholipid metabolism and sphingolipid signaling as prominent processes during early development. These findings clarify lipid partitioning between dechorionated fertilized egg contents and the chorion and provide a biochemical rationale for optimizing species-specific broodstock diets to enhance egg quality in tropical marine aquaculture.

## Simple Summary

Egg quality is a major bottleneck in marine fish aquaculture and is strongly influenced by the lipids supplied to broodstock. In this study, we compared the lipid composition of fertilized eggs (after removing the egg envelope) and the egg envelope (chorion) in three economically important tropical species—golden pompano (*Trachinotus ovatus*), longfin batfish (*Platax teira*), and leopard coral grouper (*Plectropomus leopardus*)—using high-resolution lipidomic profiling. We found clear differences among species and between tissues: egg envelopes were richer in lipids associated with structure and barrier function, whereas the dechorionated eggs contained more lipids linked to energy storage and early development. Each species showed a distinct lipid signature, indicating that broodstock of different species may require different dietary lipid formulations to consistently produce high-quality eggs. These results provide practical targets for improving broodstock nutrition and egg performance in tropical marine aquaculture and establish a reference dataset for future work on lipid-based biomarkers and egg quality management.

## 1. Introduction

Broodstock quality directly influences the survival rates of fertilized eggs and larvae in marine fish aquaculture, serving as a cornerstone for successful reproduction and seed production [1]. Lipids play a pivotal role in this process, being essential for embryonic development, yolk sac formation, and overall larval viability [2]. During vitellogenesis, essential nutrients from broodstock diets are transported into oocytes through vitellogenin and lipoprotein pathways, profoundly impacting egg size, viability, and hatching success [3]. Recent studies have highlighted the critical role of specific phospholipids and fatty acids to embryo survival and development [4,5]. However, many tropical marine fish species possess limited capacity for the de novo biosynthesis essential long-chain polyunsaturated fatty acids (LC-PUFAs) such as docosahexaenoic acid (DHA) and eicosapentaenoic acid (EPA), rendering them entirely dependent on dietary supplementation for these vital components [1].

Advancements in lipidomics have revolutionized the study of lipid dynamics in aquatic organisms, offering unprecedented insights into nutritional requirements and metabolic pathways. Non-targeted liquid chromatography-tandem mass spectrometry (LC-MS/MS)-based lipidomics facilitates the extensive characterization of diverse lipid classes, encompassing both polar species such as glycerophospholipids and non-polar compounds like triacylglycerols. Moreover, this methodology is sufficiently sensitive to reveal low-abundance functional lipids that evade detection by conventional techniques [2,6]. In fish nutrition research, lipidomics has been instrumental in delineating species-specific lipid deposition patterns during reproduction, linking dietary fatty acid profiles to egg quality and larval performance [7]. By revealing the influence of broodstock diets on hepatic lipid metabolism and ovarian lipid accumulation, integrated lipidomic analyses provide a molecular basis for refining feed formulations to enhance reproduction in marine species [8,9].

The golden pompano (*Trachinotus ovatus*), longfin batfish (*Platax teira*), and leopard coral grouper (*Plectropomus leopardus*) represent economically vital tropical marine species in Asian aquaculture, with annual production of *T. ovatus* exceeding 300 thousand tons in China alone, alongside expanding cultivation of *P. teira* for food and ornamental markets, and *P. leopardus* commanding premium prices due to its high-value traits [10]. Recent transcriptomic work has further shown that breeding temperature strongly modulates growth and nutrient metabolism in early *P. teira*, underlining the sensitivity of this species to environmental and nutritional conditions [11]. Despite their importance, these species encounter significant reproductive hurdles under natural spawning induction, where High-quality fertilized eggs typically comprise only about 30% of total spawned output in many cultured marine fishes. Among these high-quality eggs [1], hatching rates are often 60–70% at best, which ultimately constrains overall seed output and quality. This highlights the need for biochemical indicators and nutritional strategies that can improve egg quality and early developmental success [12]. To address these gaps and further elucidate lipid requirements in marine fish, the present study conducts a comparative lipidomic profiling of dechorionated fertilized eggs and chorion in these three species. The resulting dataset provides a foundation for developing targeted broodstock nutrition strategies, ultimately contributing to enhanced egg quality, larval viability, and sustainable aquaculture practices.

## 2. Materials and Methods

### 2.1. Experimental Animals and Sample Collection

Three tropical marine fish species were selected for this study: golden pompano (*Trachinotus ovatus*), longfin batfish (*Platax teira*), and leopard coral grouper (*Plectropomus leopardus*). Thereafter, abbreviated genus names (*T. ovatus*, *P. teira*, and *P. leopardus*) were used in the following text. For *T. ovatus*, broodstock consisted of 58 individuals (30 females and 28 males), with a total length of 60.4–82.3 cm. For *P. teira*, broodstock consisted of 35 individuals (20 females and 15 males), with a total length of 40.6–50.5 cm. For *P. leopardus*, broodstock consisted of 48 individuals (25 females and 23 males), with a total length of 33.4–36.1 cm. The Broodstock of golden pompano aged over 4 years from the Shenzhen Experimental Base of the South China Sea Fisheries Research Institute (Chinese Academy of Fishery Sciences, Guangzhou, Guangdong Province, China) were maintained in offshore sea cages. The Broodstock of longfin batfish aged approximately 3 years from the Hainan Experimental Base (Lingshui County, Hainan Province, China) were held in nearshore sea cages. The Broodstock of leopard coral grouper aged 2 years from the Sanya Deep-Sea Science and Technology Innovation Platform (Sanya, Hainan Province, China) were kept in nearshore sea cages. Prior to spawning induction, Broodstock were fed four times daily with wild-caught trash fish at a targeted ration of 3.0% body weight (BW) per day (i.e., 0.75% BW per feeding). Based on the estimated BW ranges (1.57–3.85 kg for *T. ovatus*; 1.44–2.74 kg for *P. teira*; 0.53–0.66 kg for *P. leopardus*), the corresponding feeding amounts were set at 20.3 g per individual per feeding for *T. ovatus*, 15.7 g per individual per feeding for *P. teira*, and 4.5 g per individual per feeding for *P. leopardus*. Nutritional fortification was provided using fish oil and vitamin supplements from the following manufacturers: for *T. ovatus*, products from Guangdong Haid Group Co., Ltd. (Guangzhou, China); for *P. teira*, products from Zhongsha Animal Health Products (Xiamen) Co., Ltd. (Xiamen, China); and for *P. leopardus*, products from Liyang Aquatic (Guangzhou, China). Spawning was induced by intramuscular administration of exogenous hormones. For *T*. *ovatus*, broodstock were injected with human chorionic gonadotropin (HCG) and Ovaprim (sGnRH-A + domperidone) at reported doses of 1000–1600 IU per body weight and 0.3–0.5 mL kg^−1^ body weight, respectively; spawning typically occurred 10–16.5 h after treatment. For *P. teira*, broodstock were anesthetized and injected intramuscularly with a hormone mixture prepared in 0.9% NaCl, with a total injection volume < 2 mL fish^−1^, containing HCG 1000 IU kg^−1^, LRH-A2 10 mg kg^−1^, and LRH-A3 10 mg kg^−1^; natural spawning generally occurred 30–36 h after induction, and fertilized eggs were collected using an 80-mesh net. For *P. leopardus*, broodstock were induced using a combination of HCG 350 IU kg^−1^ and LHRH-a 38 μg kg^−1^, and ovulation/spawning was generally observed at approximately 48 h post-injection. After spawning, buoyant fertilized eggs were collected from the spawning tanks, gently rinsed with filtered seawater, and immediately used for downstream sampling and lipidomic analysis. During broodstock holding/spawning and egg collection, filtered natural seawater was used and water-quality parameters were monitored daily. Water temperature was maintained at 26–28 °C (e.g., 26 ± 0.3 °C for *P. teira* egg incubation and 28 ± 0.3 °C for *T. ovatus* egg incubation), salinity at 30–36 ppt, and pH at 7.8–8.3. Dissolved oxygen was maintained above 5.0 mg L^−1^. Ammonia nitrogen (NH_3_–N) was controlled below 0.02 mg L^−1^ and nitrite nitrogen (NO_2_–N) was maintained below 0.10 mg L^−1^ to ensure suitable water conditions for marine fish reproduction and early development.

### 2.2. Sample Collection and Experimental Design

All samples were fertilized eggs from natural spawning and fertilization. Following natural spawning in nearshore sea cages, buoyant eggs were collected using a surface egg-collector net (200–300 μm mesh) positioned at the water outflow/surface skimming point. Egg collection was performed at 10–15 min intervals for 1–2 h after spawning. The collected eggs were immediately rinsed with filtered seawater to remove debris, and buoyant eggs were separated from sinking eggs by flotation. Fertilization was verified under a stereomicroscope by the presence of normal cleavage (e.g., 2–4 cell stage). For each spawning event, the total yield of buoyant fertilized eggs was recorded as wet mass after draining on a sieve for 1 min (*T. ovatus*: 6.5 kg; *P. teira*: 3.4 kg; *P. leopardus*: 4.6 kg per spawning event). The fertilized egg samples used for lipidomic analysis were taken from the buoyant, normally cleaving egg fraction and processed within 30 min of collection. Three biological replicates were prepared for each species.

### 2.3. Egg Envelope Isolation and Sample Preprocessing

Egg envelope (chorion) isolation was performed at 4 °C. Membrane protein extraction reagents A and B (BestBio, Shanghai, China) were dissolved and maintained on ice. Phenylmethylsulfonyl fluoride (PMSF; Sigma-Aldrich, St. Louis, MO, USA) was added immediately prior to use to achieve a final concentration of 1 mM. For each sample, 100 mg of dechorionated fertilized eggs was resuspended in prechilled phosphate-buffered saline (PBS; pH 7.4; Solarbio, Beijing, China) and centrifuged at 600× *g* for 5 min at 4 °C to pellet the cells. The supernatant was discarded, and the tube was briefly centrifuged for 1 min to remove any residual liquid. Subsequently, 1 mL of reagent A supplemented with PMSF was added, and the mixture was incubated on ice for 10–15 min for pre-lysis, followed by homogenization using a prechilled glass Dounce homogenizer (Wheaton^®^, Millville, NJ, USA) with 30–50 strokes. The number of strokes was determined based on microscopic examination, ensuring a cell disruption rate of ≥70%. The homogenate was then centrifuged at 700× *g* for 10 min at 4 °C to remove nuclei and intact cells. The supernatant was carefully collected, retaining a minimal residual volume to preserve purity. Chorion was pelleted by centrifugation at 14,000× *g* for 30 min at 4 °C and stored at −80 °C. In addition, 100 mg of dechorionated fertilized eggs from each species was snap-frozen in liquid nitrogen without egg envelope separation and stored at −80 °C for whole-egg lipidomic analysis.

### 2.4. Lipidomic Analysis

Lipidomic profiling was performed using an UltiMate 3000 UHPLC system (Thermo Fisher Scientific, Waltham, MA, USA) coupled to a Q Exactive Orbitrap mass spectrometer (Thermo Fisher Scientific, Waltham, MA, USA). Lipid extraction was conducted using a modified methanol–methyl tert-butyl ether (MTBE) protocol. Briefly, 100 mg of each sample was homogenized with 300 μL of methanol and 1 mL of MTBE in a ventilated fume hood, followed by vortex extraction for 1 h. The mixture was then transferred to a 1 mL centrifuge tube, supplemented with 300 μL of water, vortexed, and centrifuged at 12,000 rpm for 10 min at 4 °C to achieve phase separation. The upper MTBE layer (800 μL) was collected, concentrated under vacuum, and stored at −80 °C until analysis.

For chromatographic separation, samples were analyzed using a Sepax GP-C18 column (2.1 mm × 150 mm, 1.8 μm, 120 Å; Sepax Technologies, Inc., Newark, DE, USA). The column temperature was maintained at 25 °C, with a flow rate of 0.3 mL/min and an injection volume of 2 μL. In both electrospray ionization positive and negative modes, the mobile phase consisted of A = acetonitrile: water (6:4, *v*/*v*) containing 0.1% formic acid and 5 mM ammonium acetate, and B = isopropanol: acetonitrile (9:1, *v*/*v*) containing 0.1% formic acid and 5 mM ammonium acetate. The gradient elution program was set as follows: 0–1 min, 20% B; 1–11 min, 20% to 100% B (linear gradient); 11–20 min, 100% B; 20.1–30 min, 20% B for column re-equilibration. Samples were placed in a 4 °C autosampler throughout the analysis. To minimize fluctuations in instrument detection signals, a random order was used for continuous sample analysis, and quality control samples were inserted into the queue to monitor system stability and data reliability.

Mass spectrometry was performed in data-dependent acquisition mode with polarity switching. The ESI source conditions were set as follows: full MS resolution of 70,000, AGC target of 1 × 10^6^, maximum injection time of 50 ms, and scan range of 150–1500 m/z. For dd-MS^2^, the resolution was 17,500, AGC target was 1 × 10^5^, maximum IT was 50 ms, TopN was 10, and stepped normalized collision energywas 10, 30, 55.

Data preprocessing, peak alignment, and annotation were carried out using Progenesis QI (v3.0; Waters Corporation, Milford, MA, USA). Lipid identification relied on accurate mass matching (MS^1^ tolerance of 12 ppm and retention time tolerance of 0.1 min; MS^2^ tolerance of 20 ppm and RT tolerance of 0.1 min) against in-house and public databases (LIPID MAPS, KEGG). The lipidomics dataset was processed through a sequential filtering and normalization procedure. Firstly, lipids were filtered based on quantitative prevalence (retained if detected in ≥30% of samples) and qualitative reliability (excluded with a fragmentation score < 45). Furthermore, duplicate entries from LIPID MAPS were resolved by prioritizing higher fragmentation scores, or the primary score when tied. Finally, missing values were imputed using half of the minimum non-zero value in the data matrix, followed by normalization of each lipid’s abundance to the total ion intensity across all samples.

Multivariate statistical analyses, including principal component analysis (PCA) and partial least squares discriminant analysis (PLS-DA), were performed to explore lipidomic patterns. To minimize confounding by interspecific variation, two-group orthogonal partial least squares discriminant analysis (OPLS-DA) models with one orthogonal component were constructed separately for each species and validated using 200 permutation tests. Differential lipid analysis between chorion (LK) and dechorionated fertilized eggs (SJL) for each species was conducted using OPLS-DA models, with significance criteria of variable importance in projection (VIP) > 1, fold change (FC) ≥ 1.5 or ≤2/3, and false discovery rate (FDR) < 0.05 (ANOVA). Differential lipids were subjected to KEGG pathway enrichment analysis to elucidate associated biological processes; enrichment was calculated using the ratio of differential lipids mapped to each pathway, with significance determined by *p* values and corrected via FDR. All lipidomic analyses were outsourced to Beijing Baitaipark Biotechnology Co., Ltd. (Beijing, China) To avoid artefactual extreme fold changes caused by missing value imputation near the detection limit, lipids with more than 50% missing values in any group were excluded from fold-change interpretation. For the remaining lipids, very large |log_2_(FC)| values were interpreted with caution and were not used as sole evidence of biological importance; instead, emphasis was placed on pathway-level enrichment and consistent trends across related lipid species.

## 3. Results

### 3.1. Sample Data Screening and Visualization

#### 3.1.1. PCA and PLS-DA

**Figure 1:**
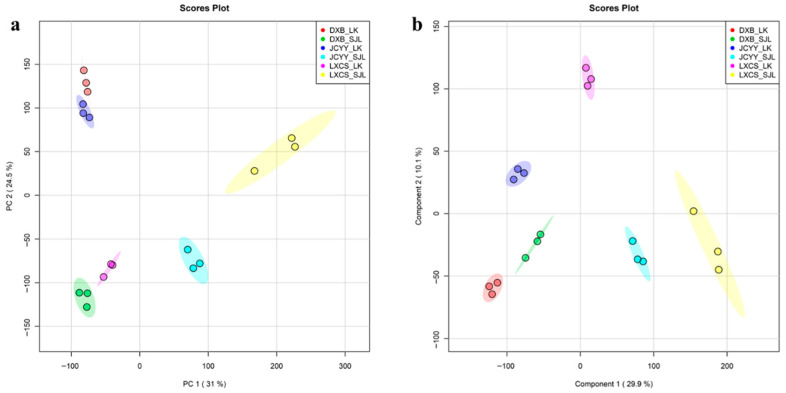
Overall lipidomic features of broodstock chorion (LK) and dechorionated fertilized eggs (SJL) in tropical marine fishes. (**a**) PCA score plot (PC1 = 31.0%, PC2 = 24.5%); (**b**) PLS-DA score plot (component 1 = 29.9%, component 2 = 10.1%).

Principal component analysis (PCA) of the 18 samples (three biological replicates each of chorion (LK) and dechorionated fertilized eggs (SJL) for the three species) revealed that PC1 and PC2 accounted for 55.5% of the total variance. The PCA score plot (Figure 1a) demonstrated interspecific separation along PC1, with dechorionated fertilized eggs of *T. ovatus* (LXCS_SJL) positioned at the far right, distinctly separated from other samples. In contrast, chorion of *P. leopardus* (DXB_LK) and *P. teira* (JCYY_LK), along with dechorionated fertilized eggs of *P. leopardus* (DXB_SJL), clustered in the negative PC1 region. PC2 primarily captured tissue-specific variation, with chorion and dechorionated fertilized eggs within each species located on opposite sides of PC2. For instance, DXB_LK exhibited higher PC2 values than DXB_SJL, and a similar pattern was observed for *P. teira*. All biological replicates clustered closely.

A supervised partial least squares discriminant analysis (PLS-DA) model (Figure 1b), based on predefined groups, showed that the first two components explained 40.0% of the variance and fully separated the six groups. Consistent with PCA, LXCS_SJL formed a distinct cluster along component 1, while within-species separation between chorion and dechorionated fertilized eggs occurred along component 2, highlighting influences of both species identity and tissue type on lipidomic profiles.

#### 3.1.2. OPLS-DA Statistics

**Figure 2:**
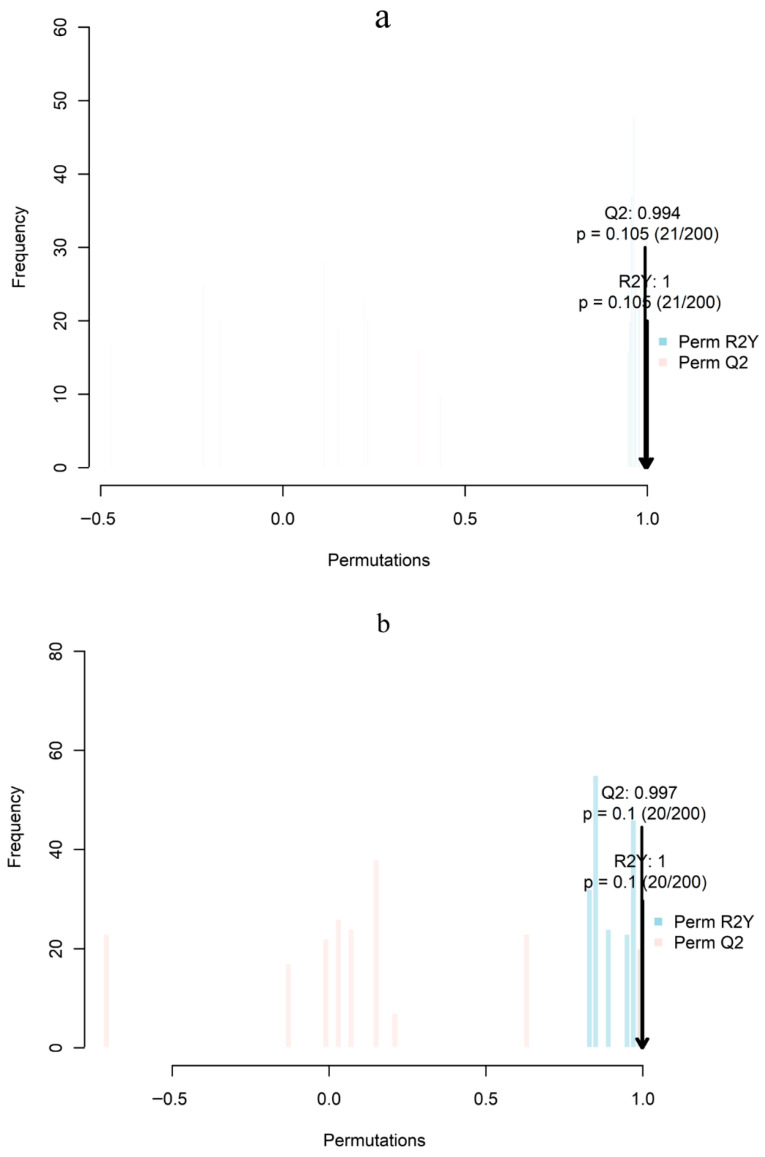

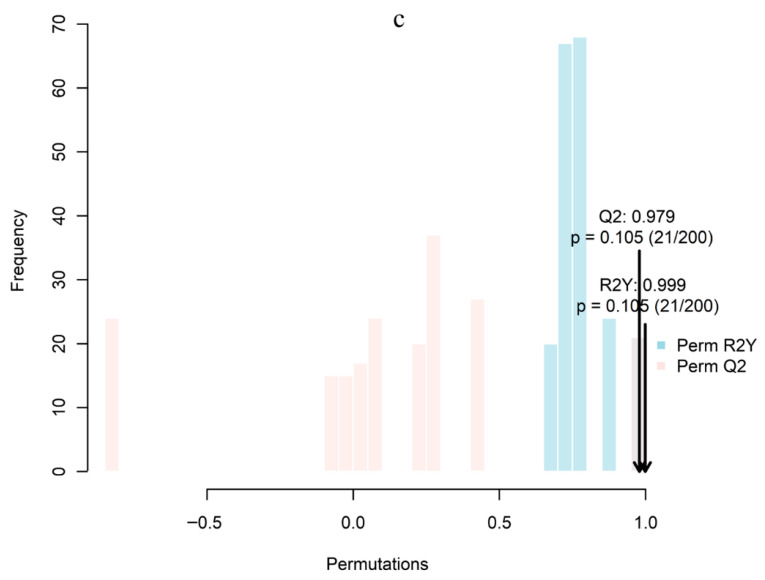
OPLS-DA model validation by 200-iteration permutation tests for chorion (LK) versus dechorionated fertilized eggs (SJL) in three fish species: (**a**) *P. leopardus* (DXB), (**b**) *P. teira* (JCYY), and (**c**) *T. ovatus* (LXCS). In all models, the original R^2^Y values were close to 1 and higher than those of the permuted distributions, supporting model validity.

To test whether systematic differences in lipid composition between chorion and dechorionated fertilized eggs were statistically supported, OPLS-DA models followed by 200 permutation tests were applied to *P. leopardus* (DXB), *P. teira* (JCYY), and *T. ovatus* (LXCS). The models demonstrated near-perfect goodness of fit (R^2^Y ≈ 1.0; DXB: 1.000; JCYY: 1.000; LXCS: 0.999) and high predictive power (Q^2^: DXB 0.994; JCYY: 0.997; LXCS: 0.979), indicating that sample type (egg envelope vs. fertilized egg) was the main driver of lipidomic separation. While the permutation test *p*-values exceeded the conventional threshold of 0.05 (0.10–0.105)—a limitation likely attributable to the small sample size (*n* = 3 per group)—the strong model fit (R^2^Y ≈ 1.0), high predictive power (Q^2^ > 0.97), and clear separation in score plots indicate biologically interpretable, tissue-specific lipid patterns. These results should therefore be considered exploratory and interpreted with due caution. Notably, the JCYY model showed the highest Q^2^, suggesting that lipid compositional differences between chorion and dechorionated fertilized eggs in *P. teira* were the most pronounced and consistent (Figure 2a–c).

#### 3.1.3. Hierarchical Clustering Heatmap

**Figure 3:**
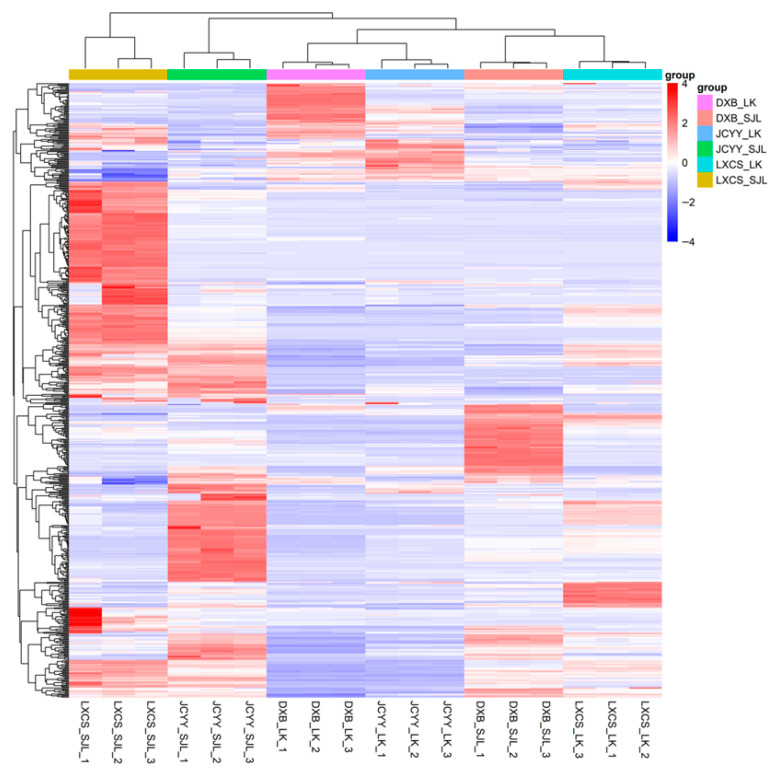
Hierarchical clustering heatmap of significantly differential lipid metabolites in positive-ion mode between chorion (LK) and dechorionated fertilized eggs (SJL) in three tropical marine fish species: DXB (*Plectropomus leopardus*), JCYY *(Platax teira*), and LXCS (*Trachinotus ovatus*). Rows represent individual lipid metabolites, columns represent samples, and the color gradient ranges from blue (low relative abundance) to red (high relative abundance). The dendrogram on the left shows the clustering of lipid metabolites based on similarity, with the top color bar indicating sample groupings.

The heatmap of differentially expressed lipids acquired in positive-ion mode revealed a clear clustering pattern: samples were primarily grouped by species, and subsequently by tissue type within each species. Several lipids displayed opposite abundance trends between chorion and dechorionated fertilized eggs. For example, dechorionated fertilized eggs of *T. ovatus* showed generally higher lipid abundance than their chorion, while in *P. teira* and *P. leopardus* certain lipids were more abundant in chorion (Figure 3).

### 3.2. Lipid Classification and Inter-Species Differences in Lipid Composition

**Figure 4:**
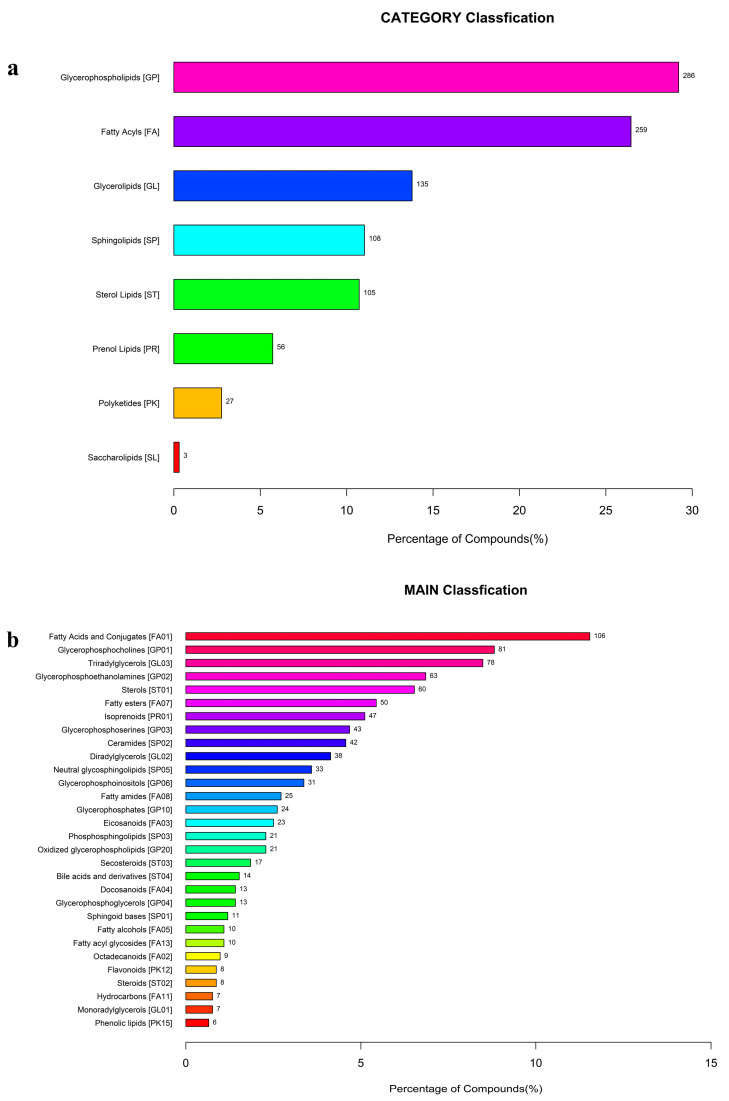
Lipid classification statistics based on the LIPID MAPS database. (**a**) Bar chart showing the percentage distribution of detected lipid compounds across the eight major categories in the combined samples from dechorionated fertilized eggs and chorion of tropical marine fish species. Categories include Glycerophospholipids (GP), Fatty Acyls (FA), Glycerolipids (GL), Sphingolipids (SP), Sterol Lipids (ST), Prenol Lipids (PR), Polyketides (PK), and Saccharolipids (SL), with bar lengths representing percentages and labels indicating compound counts. (**b**) Bar chart depicting the percentage of compounds in the top 30 lipid sub-classes derived from the detection results, ranked by abundance, such as Fatty Acids and Conjugates [FA01], Glycerophosphocholines [GP01], and Triacylglycerols [GL03], highlighting sub-class diversity within main categories. Different colors represent different lipid categories as classified according to the LIPID MAPS database.

Lipids identified across all samples were classified according to the LIPID MAPS database, encompassing eight major categories (Figure 4a): Glycerophospholipids [GP], Fatty Acyls [FA], Glycerolipids [GL], Sphingolipids [SP], Sterol Lipids [ST], Prenol Lipids [PR], Polyketides [PK], and Saccharolipids [SL]. Within these categories, the top 30 subclasses were quantified, revealing Fatty Acids and Conjugates [FA01] as the most abundant subclass at 10.6%, succeeded by Triacylglycerols [GL03] at 6.9% and Sterols [ST01] at 4.8% (Figure 4b).

To elucidate inter-species differences, the top 20 most abundant lipids in dechorionated fertilized eggs (SJL) were ranked by mean normalized intensity for each species. In *P. leopardus* (DXB_SJL), triradylglycerols [GL03] dominated, with LMGL03013423 ranked highest, followed by other GL03 lipids and sphingoid bases [SP01]. For *P. teira* (JCYY_SJL), glycerophosphoethanolamines [GP02] and glycerophosphoinositols [GP06] were prominent, led by LMGP02010339. In *T. ovatus* (LXCS_SJL), secosteroids [ST03] and fatty acids [FA01] prevailed, with LMST03020485 topping the list. These profiles underscore species-specific lipid deposition patterns, potentially reflecting distinct metabolic adaptations and nutritional requirements (Table 1).

### 3.3. Inter-Species Differences in Lipid Composition of Dechorionated Fertilized Eggs Among the Three Fish Species

#### 3.3.1. Volcano-Plot Results

Comparative analysis revealed significant lipid alterations in each species pair, with 979 lipids identified in total. Specifically, 589 lipids (189 upregulated, 400 downregulated) were differential in DXB_LK vs. DXB_SJL, 584 (89 upregulated, 495 downregulated) in JCYY_LK vs. JCYY_SJL, and 410 (147 upregulated, 263 downregulated) in LXCS_LK vs. LXCS_SJL (Table 2).

Representative differential lipids included those with substantial fold changes, such as LMFA05000647 in DXB (log_2_FC = −31.14, FC = 4.22 × 10^−10^, FDR = 1.06 × 10^−11^) and LMGL02010455 in JCYY (log_2_FC = +32.49, FC = 6.04 × 10^9^, FDR = 2.56 × 10^−10^) (Table 3).

**Figure 5:**
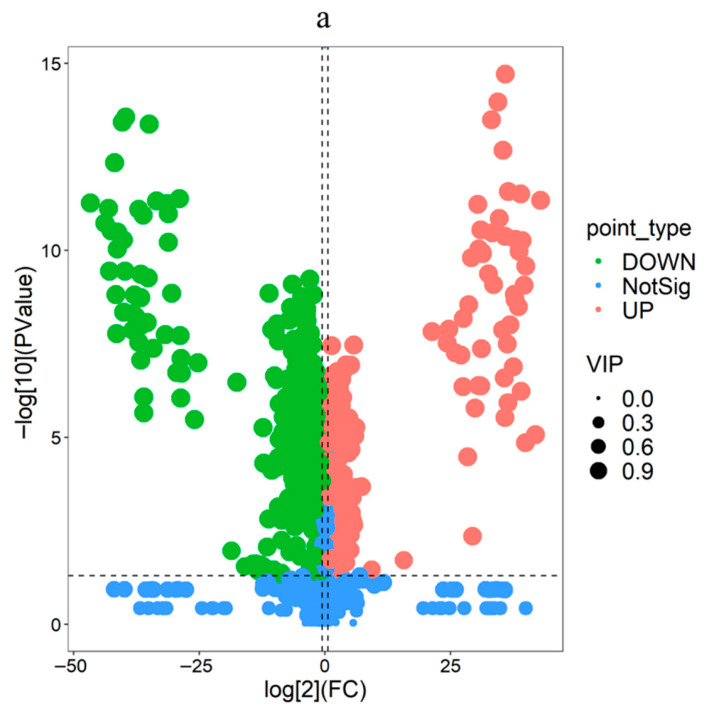

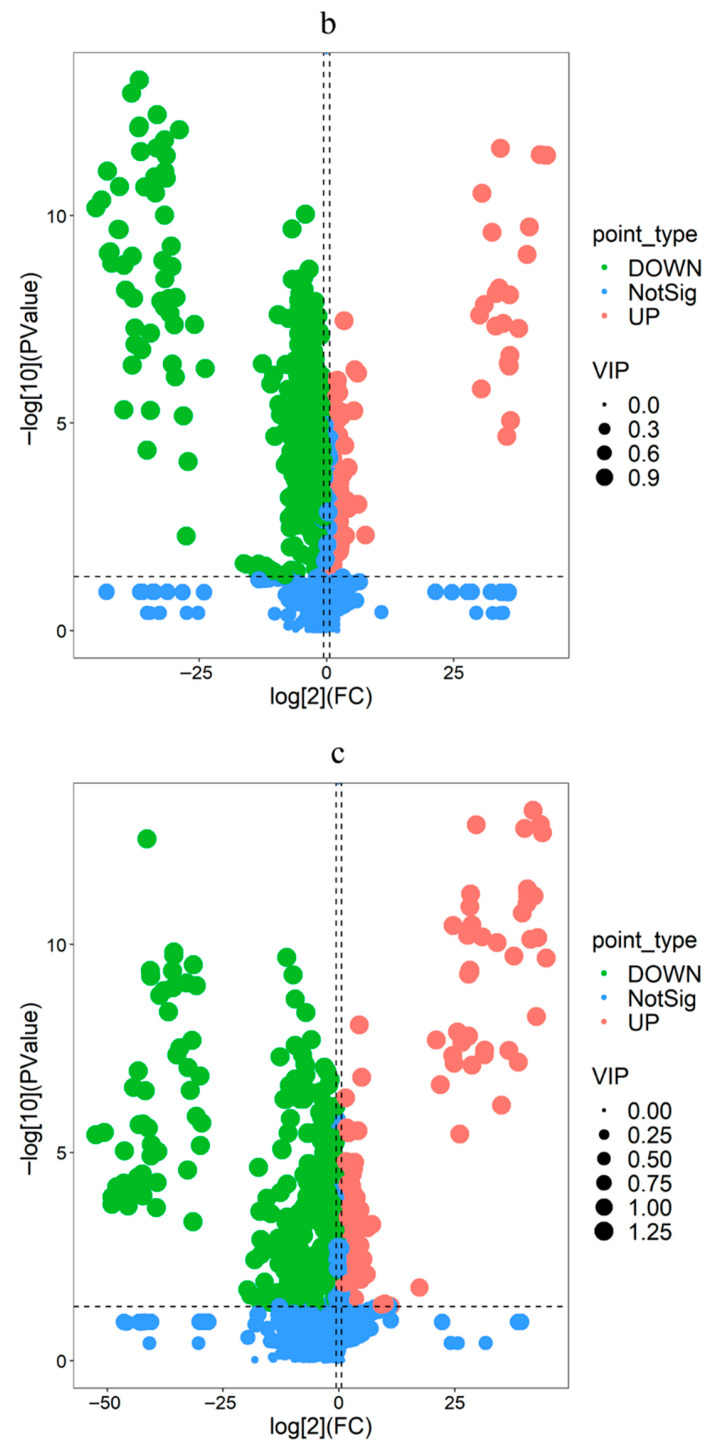
Volcano plots depicting the distribution of differential lipid metabolites between chorion (LK) and dechorionated fertilized eggs (SJL) in three tropical marine fish species. (**a**) *Plectropomus leopardus* (DXB), (**b**) *Platax teira* (JCYY), (**c**) *Trachinotus ovatus* (LXCS). Each dot represents a lipid metabolite, with the *x*-axis indicating log_2_ (Fold Change) (LK vs. SJL) and the *y*-axis indicating –log_10_ (*p*-value). Points are colored to denote regulation status: green for downregulated (DOWN), red for upregulated (UP), and blue for non-significant (NotSig). Point sizes are scaled according to Variable Importance in Projection (VIP) scores; the bubble sizes shown in the legend are provided as a visual guide to the size scale. Dashed vertical and horizontal lines indicate the fold-change threshold (|log_2_(FC)| = 1) and the statistical significance threshold (−log_10_(*p*) = 1.3, corresponding to *p* = 0.05), respectively.

Volcano plots illustrated the distribution of these differential lipids for each species (Figure 5a–c). In each plot, points represent individual lipids, with the *x*-axis denoting log_2_(FC) and the *y*-axis −log_10_(FDR). Red points indicate significantly upregulated lipids, blue points downregulated ones, and gray points non-significant lipids, highlighting pronounced tissue-specific variations in lipid profiles.

#### 3.3.2. Functional Pathway (KEGG) Analysis of Differential Lipids

**Figure 6:**
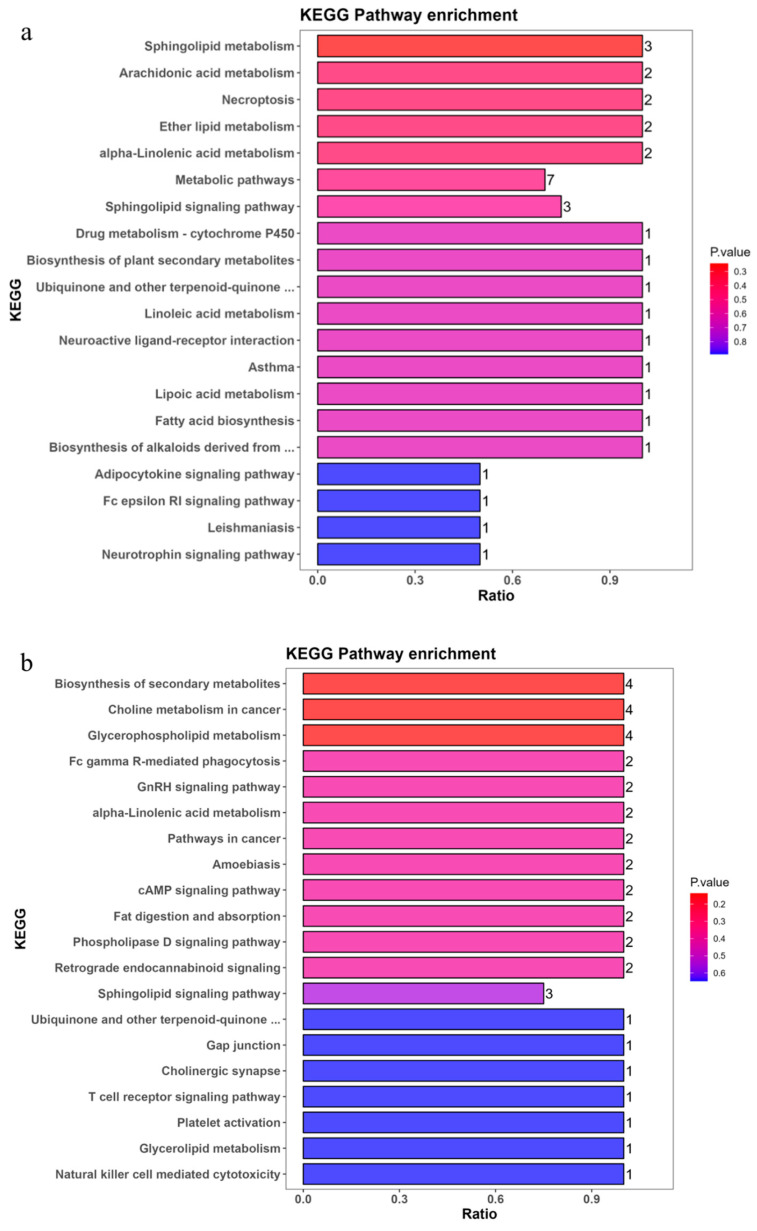

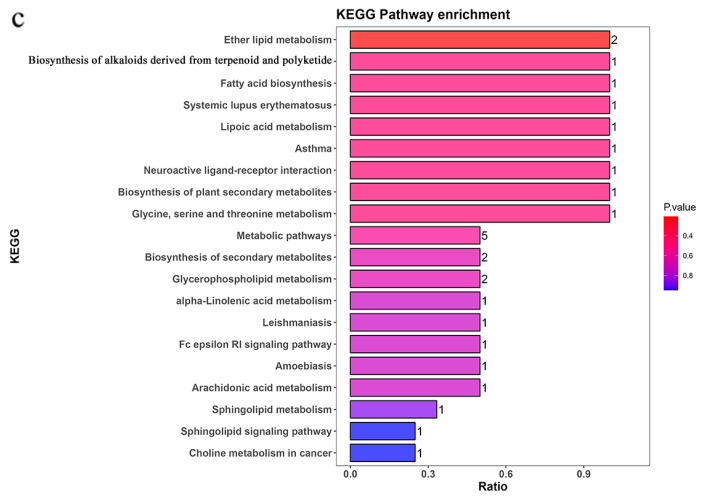
Bar graphs of metabolic pathway impact factors. The *y*-axis denotes metabolic pathways, while the *x*-axis represents the Ratio values enriched in different metabolic pathways. Colors are associated with *p* values, where redder hues indicate smaller *p* values and bluer hues indicate larger *p* values; smaller *p* values signify greater significance of the detected differential lipid metabolites’ impact on the respective pathways. (**a**) *Plectropomus leopardus*, (**b**) *Platax teira*, (**c**) *Trachinotus ovatus*.

KEGG pathway enrichment analysis demonstrated that the differential lipids between the chorion (LK) and dechorionated fertilized eggs (SJL) exhibited pronounced divergence in metabolic profiles across the three species (Figure 6a–c).

KEGG pathway enrichment analysis of *P. leopardus* (DXB) revealed distinct tiers of lipid-related pathways. The most significantly enriched pathways (*p* < 0.05) were sphingolipid metabolism (Ratio = 3), arachidonic acid metabolism, necroptosis, ether lipid metabolism, and alpha-linolenic acid metabolism (all Ratio = 2). Metabolic pathways (Ratio = 7) and the sphingolipid signaling pathway (Ratio = 3) displayed moderate enrichment. In contrast, pathways such as drug metabolism—cytochrome P450 and biosynthesis of plant secondary metabolites showed lower enrichment (Ratio = 1) with variable significance. In *P. teira* (JCYY), prominent enrichments were observed in biosynthesis of secondary metabolites (Ratio = 4), choline metabolism in cancer (Ratio = 4), and glycerophospholipid metabolism (Ratio = 4), all with high significance (red bars). Pathways such as Fc gamma R-mediated phagocytosis, GnRH signaling pathway, alpha-linolenic acid metabolism, pathways in cancer, amoebiasis, cAMP signaling pathway, fat digestion and absorption, phospholipase D signaling pathway, and retrograde endocannabinoid signaling each had a ratio of 2. Sphingolipid signaling pathway showed a ratio of 3, and less enriched pathways included ubiquinone and other terpenoid-quinone biosynthesis, gap junction, cholinergic synapse, T cell receptor signaling pathway, platelet activation, glycerolipid metabolism, and natural killer cell mediated cytotoxicity (Ratio = 1, blue bars). In *T. ovatus* (LXCS), ether lipid metabolism was the most prominently enriched pathway (Ratio = 2). Several other pathways also showed high significance (Ratio = 1), including the biosynthesis of alkaloids derived from ornithine, lysine, and nicotinic acid; fatty acid biosynthesis; systemic lupus erythematosus; lipoic acid metabolism; asthma; neuroactive ligand–receptor interaction; biosynthesis of plant secondary metabolites; and glycine, serine, and threonine metabolism. Metabolic pathways displayed the broadest involvement (Ratio = 5), while the biosynthesis of secondary metabolites and glycerophospholipid metabolism were each enriched at Ratio = 2. Additional pathways, such as alpha-linolenic acid metabolism, leishmaniasis, Fc epsilon RI signaling pathway, amoebiasis, and arachidonic acid metabolism, exhibited Ratio = 1. Sphingolipid metabolism, the sphingolipid signaling pathway, and choline metabolism in cancer were among those with lower significance.

The detailed KEGG pathway enrichment results, including *p* values, ratios, and associated differential lipids for each species, are summarized in Table 4.

Comparatively, lipid metabolism-related pathways, such as alpha-linolenic acid metabolism and sphingolipid signaling pathway, were commonly enriched across all three species, albeit with varying ratios and significance levels. Ether lipid metabolism and arachidonic acid metabolism appeared in DXB and LXCS but not prominently in JCYY, whereas choline metabolism in cancer and glycerophospholipid metabolism were notably enriched in JCYY and LXCS but absent or less significant in DXB. Overlap in signaling pathways, specifically the Fc epsilon RI signaling pathway and neuroactive ligand-receptor interaction, was observed between DXB and LXCS, while cancer-related and phagocytosis pathways were more specific to JCYY. Overall, DXB exhibited a higher emphasis on sphingolipid and arachidonic acid pathways, JCYY on secondary metabolite biosynthesis and glycerophospholipid metabolism, and LXCS on a diverse set of lipid and immune-related pathways, highlighting species-specific functional partitioning of differential lipids.

## 4. Discussion

### 4.1. Species- and Tissue-Specific Lipid Profiles in Eggs and Chorion

The lipidomic analysis revealed pronounced species- and tissue-specific variations in lipid composition between chorion (chorion) and the dechorionated fertilized eggs across the three tropical marine fish species: *T. ovatus*, *P. teira*, and *P. leopardus*. Such interspecific divergence in lipidomes is common among fishes and is detectable with modern LC-MS/MS lipidomics [2,13]. Principal component analysis (PCA) and partial least squares discriminant analysis (PLS-DA) demonstrated that PC1 primarily captured interspecies differences, accounting for 31.0% of variance, with *T. ovatus* dechorionated fertilized eggs clustering distinctly due to elevated levels of secosteroids (ST03) and fatty acids (FA01), such as LMST03020485. In contrast, *P. leopardus* dechorionated fertilized eggs were dominated by triradylglycerols (GL03), while *P. teira* showed higher glycerophosphoethanolamines (GP02) and glycerophosphoinositols (GP06). These multivariate separations and quality-related lipid signatures are consistent with recent egg lipidomics in marine broodfish [4,5]. The observed patterns are consistent with ecological adaptations of pelagic-spawning species, which are known to maintain high LC-PUFA (particularly DHA) levels to facilitate rapid neural and embryonic development—a phenomenon widely reported in studies of marine larvae and broodstock-egg systems [1,14]. For instance, pelagic eggs/larvae frequently exhibit enriched docosahexaenoic acid (DHA) to support neural growth, consistent with the highest DHA levels (10.91 ± 0.66) observed in *T. ovatus* dechorionated fertilized eggs in the present dataset [14].

Tissue-specific differences were evident along PC2 (24.5% variance), with chorion enriched in structural lipids like phosphatidylinositol (GP06) and ceramides (SP02), while dechorionated fertilized eggs contained more functional/energy lipids, including oxidized phospholipids (GP20) and triglycerides (GL03). This fits the known role of the chorion in barrier integrity and protection, versus metabolic provisioning by yolk/dechorionated fertilized eggs [3,15]. Sphingolipids (e.g., ceramides) are established structural/signaling lipids relevant to development [16]. Orthogonal PLS-DA (OPLS-DA) models confirmed these separations with high R^2^Y (≈1.0) and Q^2^ (>0.97), indicating that over two-thirds of differential lipids (e.g., 589 in *P. leopardus*, with 400 downregulated in envelopes) were upregulated in dechorionated fertilized eggs—paralleling prior egg-quality lipidomics where PL/neutral lipid partitioning tracks embryo viability [4,5]. This functional partitioning reflects the envelope’s membrane/defense role versus inner content energy metabolism and signaling; similar relationships between maternal phospholipids, neutral lipids, and egg quality have been reported in gilthead seabream reproduction systems [17]. Volcano plots further highlighted significant fold changes, such as LMFA05000647 (log_2_FC = −31.14) in *P. leopardus*, underscoring depleted structural lipids in envelopes. Hierarchical clustering heatmaps reinforced these trends, which may stem from differential yolk/inner-content utilization during embryogenesis [18].

KEGG pathway enrichment analysis of differential lipids emphasized metabolic divergences: dechorionated fertilized eggs were linked to polyunsaturated fatty acid (PUFA) metabolism (e.g., α-linolenic acid pathway, enrichment ratio 0.3636) and signaling (e.g., sphingolipid signaling, *p* < 0.001), while envelopes mapped to glycerophospholipid remodeling. The use of pathway-level interpretation for lipidomics and the developmental relevance of sphingolipid signaling are well supported [6,16]. These findings are corroborated by lipid profiling in ballan wrasse, where embryo-enriched phospholipids/PUFAs are associated with better developmental robustness and hatching outcomes [4]. Overall, the species-specific lipid deposition—highest DHA in *T. ovatus*, skewed *n*-3/*n*-6 ratio in *P. leopardus*, and lower PUFA in *P. teira*—highlights adaptive nutritional strategies and provides a baseline for targeted broodstock dietary interventions [1,5].

### 4.2. Implications for Broodstock Nutrition Based on Lipidomic Findings

The lipidomic profiles identified in this study provide actionable insights for optimizing broodstock nutrition in the three tropical marine fish species, particularly given their reliance on wild-caught trash fish, which may vary in lipid content and fatty acid composition. Interspecies differences, such as the highest DHA levels in *T. ovatus* dechorionated fertilized eggs (10.91 ± 0.66), a skewed *n*-3/*n*-6 ratio in *P. leopardus* (7.04), and generally lower PUFA in *P. teira*, suggest species-specific dietary adjustments to enhance egg quality and larval survival. For *T. ovatus*, the enrichment of DHA in dechorionated fertilized eggs aligns with requirements for rapid neural development in pelagic species, as evidenced by studies on related pompano species where dietary DHA at approximately 15% of total fatty acids in broodstock feeds improved larval stress resistance and reduced deformities [19,20]. Consistently, dietary inclusion of the DHA-rich microalga Aurantiochytrium has been shown to enhance growth and immune indices in *T. ovatus*, further underscoring the species’ dependence on adequate *n*-3 LC-PUFA supply [21]. To address this, supplementing trash fish diets with fish oil sources rich in DHA (e.g., to achieve 10–15% crude lipid with DHA comprising 8–12% of total fatty acids) could ensure consistent deposition, preventing deficiencies observed in the lower PUFA groups.

The imbalanced *n*-3/*n*-6 ratio in *P. leopardus* suggests a potential ARA deficiency, which is crucial for prostaglandin synthesis and inflammation regulation in embryogenesis. This finding is consistent with marine fish studies demonstrating that an optimal ARA/EPA ratio (e.g., around 0.47) supports improved growth and lipid metabolism while maintaining reproductive function [22]. Recommendations include blending trash fish with moderate *n*-6 sources, such as fungal oils or vegetable oils (e.g., soybean oil at 1–2% inclusion), to balance the *n*-3/*n*-6 ratio to 4–5, thereby improving inner egg content functionality as seen in the differential lipid analysis (e.g., 189 upregulated lipids in dechorionated fertilized eggs).

The low PUFA levels observed in the dechorionated fertilized eggs of *P. teira* suggest insufficient maternal transfer from broodstock diets. This aligns with findings in freshwater fish such as Nile tilapia (*Oreochromis niloticus*), in which broodstock diets formulated at ~10% crude lipid have been reported to improve reproductive performance and larval quality [23]. However, marine finfish such as golden pompano (*Trachinotus ovatus*) and gilthead seabream (*Sparus aurata*) typically have a greater demand for HUFAs, indicating that these baseline requirements may need to be adjusted upward for optimal results [24]. Enhancing crude lipid to 10–15% through enriched feeds could boost PUFA accumulation, as demonstrated in gilthead seabream where high *n*-3 LC-PUFA diets (e.g., EPA 14.97%, DHA 15.56% of total fatty acids) significantly increased egg viability and larval production [25]. Across species, maintaining a DHA:EPA:ARA ratio of approximately 1:0.5:0.2, as inferred from the lipidomic data and supported by these studies, would optimize functional lipid partitioning between chorion and dechorionated fertilized eggs, ultimately improving reproductive outcomes without excess fat accumulation.

### 4.3. Applications and Limitations

This study provides an exploratory, untargeted comparison of lipidomic profiles between chorion and dechorionated fertilized eggs in three tropical marine fish species. Several limitations should be noted. First, the biological replicate size was limited (*n* = 3 per group), which reduces statistical power and may increase both false negatives and uncertainty in multivariate validation. Second, the untargeted workflow mainly yields relative abundances rather than absolute concentrations; therefore, key lipid candidates identified here should be verified by targeted LC–MS/MS using authentic standards and appropriate internal standards. Third, confident structural assignment remains challenging for many lipid classes due to isomerism (e.g., acyl-chain position/double-bond isomers), and thus some annotations should be interpreted at the class/subclass level unless further confirmed by higher-resolution MS/MS strategies. Finally, the present findings are correlative and do not establish causality between lipid features and reproductive outcomes; future studies should integrate controlled broodstock nutritional manipulations and functional endpoints (e.g., fertilization rate, embryo survival and early larval performance) to validate biological relevance [25]. Beyond broodstock nutrition and reproductive physiology, these lipidomic baselines may also inform future hypothesis-driven studies on biocompatible nanoparticle-based delivery strategies for early embryos; however, such applications require dedicated validation and are beyond the scope of the present study.

## 5. Conclusions

This study has demonstrated pronounced species- and tissue-specific variations in lipid profiles between chorion and dechorionated fertilized eggs in *T. ovatus*, *P. teira*, and *P. leopardus*, addressing the research aims of elucidating lipidomic differences and informing broodstock nutrition in tropical marine fishes. Key findings reveal interspecies divergences, such as elevated DHA in *T. ovatus* dechorionated fertilized eggs for neural support, imbalanced *n*-3/*n*-6 ratios in *P. leopardus* indicating ARA needs, and lower PUFA in *P. teira* suggesting enhanced dietary HUFA, which collectively underscore adaptive metabolic strategies and provide a novel baseline for precision feed formulations to boost egg quality and larval survival. These insights extend beyond nutritional science, revealing the potential of lipid-mimicking nanoparticles for CRISPR-based editing of traits such as disease resistance and underscoring the utility of lipidomics in traceability and sustainability. Furthermore, current limitations—including small sample sizes and reliance on relative quantification—hinder broader applicability, highlighting a pressing need for standardized protocols. Consequently, future work should employ larger, multi-omics studies coupled with dietary interventions to validate lipid profiles, refine broodstock management, and pave the way for LNP applications across diverse marine species.

## Figures and Tables

**Table 1:** Top 20 lipid molecules ranked by relative abundance in dechorionated fertilized eggs of leopard coral grouper *Plectropomus leopardus* (DXB), golden pompano *Trachinotus ovatus* (LXCS), and longfin batfish *Platax teira* (JCYY), respectively.

*Plectropomus leopardus* (DXB)		*Platax teira* (JCYY)		*Trachinotus ovatus* (LXCS)	
LIPID_ID	Main Class	LIPID_ID	Main Class	LIPID_ID	Main Class
LMGL03013423	Triradylglycerols [GL03]	LMGP02010339	Glycerophosphoethanolamines [GP02]	LMST03020485	Secosteroids [ST03]
LMGL03013510	Triradylglycerols [GL03]	LMGP06050026	Glycerophosphoinositols [GP06]	LMFA01090025	Fatty Acids and Conjugates [FA01]
LMGL03013401	Triradylglycerols [GL03]	LMSP05010085	Neutral glycosphingolipids [SP05]	LMPK11000022	Cytochalasins [PK11]
LMGL03013828	Triradylglycerols [GL03]	LMFA07090170	Fatty esters [FA07]	LMFA08020091	Fatty amides [FA08]
LMSP01040011	Sphingoid bases [SP01]	LMGP01010586	Glycerophosphocholines [GP01]	LMST01130061	Sterols [ST01]
LMPK15050009	Phenolic lipids [PK15]	LMGP01011834	Glycerophosphocholines [GP01]	LMST01010568	Sterols [ST01]
LMGL03014302	Triradylglycerols [GL03]	LMSP02010024	Ceramides [SP02]	LMPK04000026	Macrolides and lactone polyketides [PK04]
LMST01031228	Sterols [ST01]	LMGP03010475	Glycerophosphoserines [GP03]	LMST01130021	Sterols [ST01]
LMFA01150073	Fatty Acids and Conjugates [FA01]	LMGP06010012	Glycerophosphoinositols [GP06]	LMGP10050020	Glycerophosphates [GP10]
LMFA08020242	Fatty amides [FA08]	LMSP0501AB03	Neutral glycosphingolipids [SP05]	LMPK12020242	Flavonoids [PK12]
LMST01040212	Sterols [ST01]	LMGP06010932	Glycerophosphoinositols [GP06]	LMSP05010092	Neutral glycosphingolipids [SP05]
LMFA01050079	Fatty Acids and Conjugates [FA01]	LMGP02010004	Glycerophosphoethanolamines [GP02]	LMPR01070459	Isoprenoids [PR01]
LMGP10030007	Glycerophosphates [GP10]	LMGP06050012	Glycerophosphoinositols [GP06]	LMFA01050074	Fatty Acids and Conjugates [FA01]
LMST01100034	Sterols [ST01]	LMGP01050012	Glycerophosphocholines [GP01]	LMSP03020013	Phosphosphingolipids [SP03]
LMFA01050215	Fatty Acids and Conjugates [FA01]	LMGP06010437	Glycerophosphoinositols [GP06]	LMST01170001	Sterols [ST01]
LMPR0103110002	Isoprenoids [PR01]	LMGP06010013	Glycerophosphoinositols [GP06]	LMST05050007	Steroid conjugates [ST05]
LMFA01010019	Fatty Acids and Conjugates [FA01]	LMGP01050047	Glycerophosphocholines [GP01]	LMST03020303	Secosteroids [ST03]
LMPK15040005	Phenolic lipids [PK15]	LMGP01050048	Glycerophosphocholines [GP01]	LMFA01030338	Fatty Acids and Conjugates [FA01]
LMST01040209	Sterols [ST01]	LMGP01050050	Glycerophosphocholines [GP01]	LMGP10050033	Glycerophosphates [GP10]
LMST03020132	Secosteroids [ST03]	LMGL02010102	Diradylglycerols [GL02]	LMFA01060138	Fatty Acids and Conjugates [FA01]

**Table 2:** Summary of differentially abundant lipids identified between chorion (LK) and dechorionated fertilized eggs (SJL) for each of the three tropical marine fish species.

Compared Samples	Total	Total Sig	Sig Proportion (%)	Up	Up Proportion (%)	Down	Down Proportion (%)	Up/Down Ratio
DXB_LK vs. DXB_SJL	979	589	60.2	189	32.1	400	67.9	1:2.1
JCYY_LK vs. JCYY_SJL	979	584	59.7	89	15.2	495	84.8	1:5.6
LXCS_LK vs. LXCS_SJL	979	410	41.9	147	35.9	263	64.1	1:1.8

**Table 3:** Representative differential lipids with fold changes and significance.

Species	Lipid_ID	Lipid Class	log_2_FC	FC	FDR	−log_10_ FDR
DXB	LMFA05000647	Fatty alcohols [FA05]	−31.14	4.22 × 10^−10^	1.06 × 10^−11^	10.97
DXB	LMGP20070028	Oxidized glycerophospholipids [GP20]	−31.18	4.11 × 10^−10^	6.14 × 10^−11^	10.21
DXB	LMGL03013510	Triradylglycerols [GL03]	−34.96	2.99 × 10^−11^	4.25 × 10^−14^	13.37
DXB	LMGP06050013	Glycerophosphoinositols [GP06]	+35.80	5.97 × 10^10^	4.18 × 10^−11^	10.38
DXB	LMST05030001	Steroid conjugates [ST05]	−37.04	7.05 × 10^−12^	8.01 × 10^−12^	11.10
DXB	LMPR01070692	Isoprenoids & derivatives [PR01]	+35.84	6.13 × 10^10^	1.95 × 10^−15^	14.71
DXB	LMFA07090046	Fatty acid esters [FA07]	+34.36	2.20 × 10^10^	1.09 × 10^−14^	13.96
DXB	LMGP02010865	Glycerophosphoethanolamines [GP02]	+33.12	9.31 × 10^9^	3.22 × 10^−14^	13.49
DXB	LMPR01070109	Isoprenoids & derivatives [PR01]	+35.36	4.42 × 10^10^	2.14 × 10^−13^	12.67
DXB	LMGP06010361	Glycerophosphoinositols [GP06]	+42.88	8.09 × 10^12^	4.67 × 10^−12^	11.33
JCYY	LMGL02010455	Diradylglycerols [GL02]	+32.49	6.04 × 10^9^	2.56 × 10^−10^	9.59
JCYY	LMFA01050537	Fatty acids & conjugates [FA01]	+30.53	1.55 × 10^9^	2.90 × 10^−11^	10.54
JCYY	LMFA07050428	Fatty acid esters [FA07]	−4.14	5.69 × 10^−2^	9.35 × 10^−11^	10.03
JCYY	LMGP03050022	Glycerophosphoserines [GP03]	−6.80	8.95 × 10^−3^	2.10 × 10^−10^	9.68
JCYY	LMSP02040005	Ceramides [SP02]	+42.00	4.39 × 10^12^	3.41 × 10^−12^	11.47
JCYY	LMPR01070810	Isoprenoids & derivatives [PR01]	−40.76	5.36 × 10^−13^	2.18 × 10^−10^	9.66
JCYY	LMPR01070304	Isoprenoids & derivatives [PR01]	−31.82	2.65 × 10^−10^	1.51 × 10^−12^	11.82
JCYY	LMSP0502AB01	Neutral glycosphingolipids [SP05]	−42.53	1.57 × 10^−13^	7.42 × 10^−10^	9.13
JCYY	LMFA01030599	Fatty acids & conjugates [FA01]	−31.51	3.27 × 10^−10^	3.62 × 10^−12^	11.44
JCYY	LMGP20020058	Oxidized glycerophospholipids [GP20]	−42.89	1.23 × 10^−13^	8.07 × 10^−10^	9.09
LXCS	LMGL01010034	Monoradylglycerols [GL01]	−2.17	2.22 × 10^−1^	2.42 × 10^−7^	6.62
LXCS	LMSP02010223	Ceramides [SP02]	−5.91	1.67 × 10^−2^	1.96 × 10^−8^	7.71
LXCS	LMST04050011	Steroid conjugates [ST04]	−2.63	1.61 × 10^−1^	1.10 × 10^−7^	6.96
LXCS	LMGP03050019	Glycerophosphoserines [GP03]	−30.74	5.58 × 10^−10^	9.89 × 10^−10^	9.00
LXCS	LMGP03050013	Glycerophosphoserines [GP03]	−9.46	1.42 × 10^−3^	2.67 × 10^−8^	7.57
LXCS	LMSP05010043	Neutral glycosphingolipids [SP05]	−35.76	1.72 × 10^−11^	1.10 × 10^−9^	8.96
LXCS	LMFA13040156	Fatty acid amides [FA13]	+4.41	2.13 × 10^1^	8.65 × 10^−9^	8.06
LXCS	LMGP01050140	Glycerophosphocholines [GP01]	−9.89	1.05 × 10^−3^	5.42 × 10^−10^	9.27
LXCS	LMGP01050056	Glycerophosphocholines [GP01]	−9.39	1.49 × 10^−3^	2.07 × 10^−9^	8.68
LXCS	LMST04070031	Steroid conjugates [ST04]	−1.94	2.60 × 10^−1^	7.96 × 10^−7^	6.10

**Table 4:** KEGG pathway enrichment analysis of differential lipids.

Pathway ID	Pathway	Ratio	*p* Value	Enrichment	FDR
path:map05231	Choline metabolism in cancer	0.363636	0.000000	7.898393	0.000001
path:map04071	Sphingolipid signaling pathway	0.266666	0.000000	7.284695	0.000003
path:map00564	Glycerophospholipid metabolism	0.076923	0.000010	5.014353	0.000322
path:map00600	Sphingolipid metabolism	0.12	0.000038	4.420367	0.000950
path:map04722	Neurotrophin signaling pathway	0.4	0.000070	4.151968	0.001409

## Data Availability

The original contributions presented in this study are included in the article. Further inquiries can be directed to the corresponding authors.

## Abbreviations

The following abbreviations are used in this manuscript:

LK	chorion
SJL	dechorionated fertilized eggs
DXB	*Plectropomus leopardus*
JCYY	*Platax teira*
LXCS	*Trachinotus ovatus*
FA	Fatty Acyls
GL	Glycerolipids
GP	Glycerophospholipids
PK	Polyketides
PR	Prenol Lipids
SL	Saccharolipids
SP	Sphingolipids
ST	Sterol Lipids

## References

[1] Thiruvasagam T., Chidambaram P., Ranjan A., Komuhi N. (2024). Significance of fatty acids in fish broodstock nutrition. Anim. Reprod. Sci..

[2] Rey F., Melo T., Lopes D., Couto D., Marques F., Domingues M.R. (2022). Applications of lipidomics in marine organisms: Progress, challenges and future perspectives. Mol. Omics.

[3] Birk D.S., Onose S., Kinoshita M., Murata K. (2022). Medaka, *Oryzias latipes*, chorion are created by ovarian-expressed ZP proteins and liver-expressed choriogenins. Zool. Lett..

[4] Malzahn A., Sarno A., Hagemann A., Farkas J., Musialak L.A., Kjørsvik E., Hansen B.H. (2022). Can lipidomics help identifying egg quality in ballan wrasse?. Aquaculture.

[5] Hansen B., Kjørsvik E., Malzahn A.M., Sarno A., Kulild O.M., Farkas J., Nordtug T., Rye R., Kvæstad B., Lein I. (2022). Ova lipid profiling and egg quality in wild and captive lumpfish, *Cyclopterus lumpus* (Linnaeus, 1758). Aquaculture.

[6] Rakusanova S., Cajka T. (2024). Tips and tricks for LC–MS-based metabolomics and lipidomics analysis. TrAC Trends Anal. Chem..

[7] Tietel J., Rak G., Nogueira J.N. (2023). An overview of food lipids toward food lipidomics. Compr. Rev. Food Sci. Food Saf..

[8] Wang G., Zeng X., Li K., Liu R., Jiang Z., Ba X., Wei Z., Liu J., Liu L. (2025). Transcriptomics-driven lipidomics reveal the hepatic nutrition and lipid metabolism during ovarian development of *Conger myriaster*. Aquaculture.

[9] Wang J., Chen R., Xie Y., Qin X., Zhou Y., Xu C. (2025). Endo/lysosomal-escapable lipid nanoparticle platforms for enhancing mRNA delivery in cancer therapy. Pharmaceutics.

[10] Nyunoya T., Noda T., Kawamoto Y., Hayashi Y., Ishibashi Y., Ito M., Okino N. (2021). Lack of ∆5 Desaturase Activity Impairs EPA and DHA Synthesis in Fish Cells from Red Sea Bream and Japanese Flounder. Mar. Biotechnol..

[11] Liu M.-J., Gao J., Guo H.-Y., Zhu K.-C., Liu B.-S., Zhang N., Sun J.-H., Zhang D.-C. (2023). Transcriptomics Reveal the Effects of Breeding Temperature on Growth and Metabolism in the Early Developmental Stage of *Platax teira*. Biology.

[12] Williams K.C., Irie T., Cadoret J.-P. (2019). Recent advances of marine ornamental fish larviculture: Broodstock reproduction, live prey and feeding regimes, and comparison between demersal and pelagic spawners. Rev. Aquac..

[13] Cao X., Xu M., Feng T., Li R., Song Y., Meng N., Fan X., Zeng J., Xu J. (2024). A comparative lipid profile of four fish species: From muscle to industrial by-products based on RPLC−Q-TOF-MS/MS. Food Res. Int..

[14] Koven W., Yanowski E., Gardner L., Nixon O., Block B. (2024). Docosahexaenoic acid (DHA) is a driving force regulating gene expression in bluefin tuna (*Thunnus thynnus*) larval development. Sci. Rep..

[15] Valdebenito I., Pérez-Atehortúa M., Hernández A.J., Dantagnan P., Silva M., Risopatrón J., Farías J., Villalobos E.F., Valdebenito I. (2023). Chorion in fish: Synthesis, functions and factors associated with its malformations. Aquac. Rep..

[16] Kuo A., Hla T. (2024). Regulation of cellular and systemic sphingolipid homeostasis. Nat. Rev. Mol. Cell Biol..

[17] Ferosekhan S., Xu H., Turkmen S., Gómez A., Afonso J.M., Fontanillas R., Rosenlund G., Kaushik S., Izquierdo M. (2020). Reproductive performance of gilthead seabream (*Sparus aurata*) broodstock showing different expression of fatty acyl desaturase 2 and fed two dietary fatty acid profiles. Sci. Rep..

[18] Xu M., Legradi J., Leonards P. (2022). Using comprehensive lipid profiling to study effects of PFHxS during different stages of early zebrafish development. Sci. Total Environ..

[19] Mejri S.C., Tremblay R., Audet C., Wills P.S., Riche M. (2021). Essential fatty acid requirements in tropical and cold-water marine fish larvae and juveniles. Front. Mar. Sci..

[20] Fu Z., Yang R., Zhou S., Ma Z., Zhang T. (2021). Effects of rotifers enriched with different enhancement products on larval performance and jaw deformity of golden pompano larvae *Trachinotus ovatus* (Linnaeus, 1758). Front. Mar. Sci..

[21] Li S., Wang B., Liu L., Song Y., Lv C., Zhu X., Luo Y., Cheng C.H.K., Chen H., Yang X. (2020). Enhanced Growth Performance Physiological and Biochemical Indexes of *Trachinotus ovatus* Fed with Marine Microalgae *Aurantiochytrium* sp. Rich in n-3 Polyunsaturated Fatty Acids. Front. Mar. Sci..

[22] Magalhães R., Guerreiro I., Santos R.A., Coutinho F., Couto A., Serra C.R., Olsen R.E., Peres H., Oliva-Teles A. (2020). Oxidative status and intestinal health of gilthead sea bream (*Sparus aurata*) juveniles fed diets with different ARA/EPA/DHA ratios. Sci. Rep..

[23] Ng W.-K., Wang Y. (2011). Inclusion of crude palm oil in the broodstock diets of female Nile tilapia, *Oreochromis niloticus*, resulted in enhanced reproductive performance compared to broodfish fed diets with added fish oil or linseed oil. Aquaculture.

[24] Engdaw F., Geremew A. (2024). Broodstock nutrition in Nile tilapia and its implications on reproductive efficiency. Front. Aquac..

[25] Gerichten J., Saunders K., Bailey M.J., Gethings L.A., Onoja A., Geifman N., Spick M. (2024). Challenges in lipidomics biomarker identification: Avoiding the pitfalls and improving reproducibility. Metabolites.

